# A congeneric and non-randomly associated pair of larval trematodes dominates the assemblage of co-infecting parasites in fathead minnows (*Pimephales promelas*)

**DOI:** 10.1017/S0031182023000859

**Published:** 2023-09

**Authors:** Sarah V. Hirtle, Sangwook Ahn, Cameron P. Goater

**Affiliations:** Department of Biological Sciences, University of Lethbridge, AB, Lethbridge, Canada

**Keywords:** co-infection, digenean, metacercariae, multiparasitism, *Ornithodiplostomum*, parasite intensity, *Posthodiplostomum*, trematode

## Abstract

Individual hosts are often co-infected with multiple parasite species. Evidence from theoretical and empirical studies supports the idea that co-occurring parasites can impact each other and their hosts *via* synergistic or antagonistic interactions. The fundamental aim of understanding the consequences of co-infection to hosts and parasites requires an understanding of patterns of species co-occurrence within samples of hosts. We censused parasite assemblages in 755 adult, male fathead minnows collected from 7 lakes/ponds in southern Alberta, Canada between 2018 and 2020. Fifteen species of endoparasites infected fathead minnows, 98% of which were co-infected with between 2 and 9 parasite species (mean species richness: 4.4 ± 1.4). Non-random pairwise associations were detected within the overall parasite community. There were particularly strong, positive associations in the occurrences and intensities of the 2 congeneric larval trematodes *Ornithodiplostomum* sp. and *Ornithodiplostomum ptychocheilus* that comprised >96% of the 100 000+ parasites counted in the total sample of minnows. Furthermore, the occurrence of *Ornithodiplostomum* sp. was a strong predictor of the occurrence of *O. ptychocheilus*, and vice versa. Positive covariation in the intensities of these 2 dominants likely arises from their shared use of physid snails as first intermediate hosts in these waterbodies. These 2 species represent a predictable and non-random component within the complex assemblage of parasites of fathead minnows in this region.

## Graphical abstract

**Figure:**
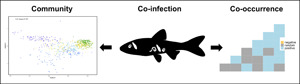

## Introduction

Individual hosts are often simultaneously infected with multiple parasite species. These multi-species infections are considered the rule, rather than the exception, for parasite assemblages in humans, domestic animals, and wildlife (Petney and Andrews, 1998; Bordes and Morand, 2011). The results of studies completed over the past 2 decades indicate that co-occurring parasites can profoundly impact each other and their hosts. For example, co-infecting parasites alter host susceptibility to infection and infection duration (Telfer *et al*., 2010; Clerc *et al*., 2019), disease progression and severity (Graham *et al*., 2005; Lamb *et al*., 2005) and patterns of parasite-induced host mortality (Thumbi *et al*., 2014). Co-infection may also influence the success of parasite control strategies (Lello *et al*., 2004). Expanding our knowledge of the nature of interactions that occur between co-infecting parasites in wild hosts is critical for a paradigm shift towards multiparasitism in disease ecology, epidemiology and veterinary medicine.

Despite increased research focus on the patterns and processes of parasite co-infection, our understanding of co-infection in wild animal hosts is limited. Even for vertebrate taxa with comparatively well-studied parasite communities such as those in teleost fish, there is a paucity of data on the incidence and consequences of co-infection (Chapman *et al*., 2021). A second fundamental question surrounds the strength, direction and repeatability of the variation that characterizes interspecific parasite species' co-occurrences and intensities. Pairwise associations between species are often inconsistent across space and time (Dezfuli *et al*., 2001; Poulin and Valtonen, 2002) and consequently, intra- and interspecific interactions are not considered a major structuring influence for parasite communities in fish (Kennedy, 2009). Resolution of these knowledge gaps requires a host–parasite system that permits analyses of patterns of co-infection over broad spatial and temporal scales and one that is amenable to experimental manipulation.

The fathead minnow (*Pimephales promelas*) is a small-bodied cyprinid fish that is abundant and widely distributed across much of the North American continent (Scott and Crossman, 1973). Fatheads and to a lesser extent brook stickleback, *Culea inconstans*, often dominate fish communities in the shallow, productive lakes and wetlands that characterize the Prairie Pothole Region of the northern Great Plains (Herwig *et al*., 2010). The parasite species that infect the fathead minnow throughout its wide geographical range in Canada have been previously reported (McDonald and Margolis, 1995). Host survey results indicate that the parasite assemblage of fatheads collected from lentic habitats tend to be dominated by larval strigeoid trematodes such as the brain- and liver-encysting trematodes *Ornithodiplostomum ptychocheilus* and *Posthdiplostomum minimum*, respectively. Among this complex suite of aquatic parasites, it is clear that co-infection is common (Sandland *et al*., 2001; Wisenden *et al*., 2012). However, patterns of co-infection and their consistency over broad spatial and temporal scales are unknown in this system.

The purpose of this study is to characterize variation in patterns of parasite species co-infection in fathead minnows sampled from lentic waterbodies in southern Alberta, Canada between 2018 and 2020. Our first aim is to utilize parasite count data from fully censused individual hosts (Bush *et al*., 1997) to characterize spatiotemporal variation in interspecific occurrences (prevalence of individual species within a sample of hosts) and mean abundances (average numbers of parasites per species within a sample of hosts). The main purpose of this description of community-level patterns is to identify sets of species that regularly co-infect individual minnows and to evaluate whether patterns of co-infection are spatially and temporally consistent. We then determine the strength and direction of pairwise associations from patterns of species co-occurrence to detect particular species pairs that form predictable, non-random components of the community.

## Materials and methods

### Host collection

**Figure 1.**
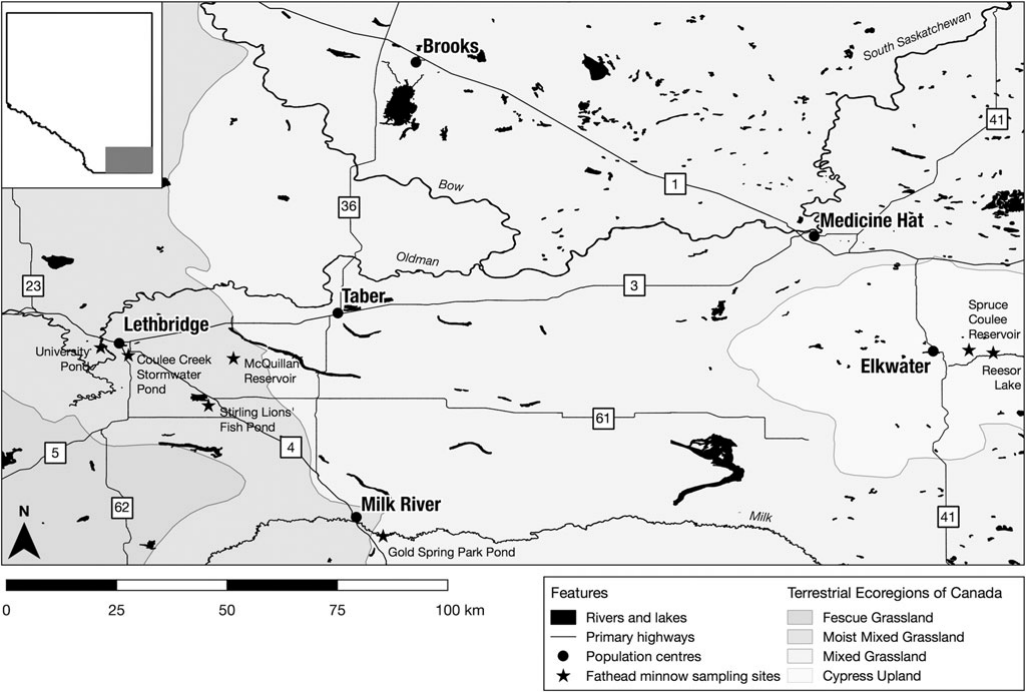
Sample locations for the collection of fathead minnows (*Pimephales promelas*) in southern Alberta. Sampling sites are indicated by star shapes.

We sampled fathead minnows from 7 small- to medium-sized lakes/ponds in southern Alberta, Canada (Fig. 1; Table S1) as part of our ongoing survey of minnows and their parasites in the region. These sites are broadly representative of lentic waterbodies in southern Alberta, where the adjacent mixed grass prairie has been substantially altered for residential and recreational development and cropland production. All sites are artificial waterbodies maintained by stormwater runoff, damming or irrigation water diversion.

Sampling took place in late May and early-to-mid June between 2018 and 2020. We collected sexually mature, male minnows (age-2) to emphasize spatial and annual variation in parasite prevalence and abundance in the absence of confounding factors such as season, host age and host sex. Minnows were caught with un-baited Gee traps set for 2–24 h in approximately 50 cm of water at a distance of 2 m from the shoreline. At the time of collection, we identified mature male minnows by the presence of breeding tubercles on the snout. Minnows were transported to the University of Lethbridge in aerated coolers, where sex and maturity were later confirmed by the presence of fully developed testes at necropsy.

### Parasite community census

A total of 755 adult, male minnows were collected over the consecutive 3-year period from the 7 sites. Fish were euthanized in the laboratory *via* cervical dislocation and then examined fresh. Minnows were measured for fork and standard lengths (mm) then weighed to the nearest 0.01 g. Parasite infracommunities were fully censused using standard necropsy techniques. We first scanned fish for melanized, larval trematodes encysted in the epidermis and associated musculature. Next, we assessed the olfactory chambers for the monogenean *Dactylogyrus olfactorius* (Lari *et al*., 2016). We then removed the brain, gut and viscera, placed the tissue between 2 glass plates, and examined the preparation for larval and adult helminths under a stereomicroscope. The eyes were dissected to separate the lens and humour for assessment of larval strigeoid trematodes. Following Marcogliese *et al*. (2001), we recorded all unencysted metacercariae from the eye lenses as *Diplostomum* sp. Finally, we removed the spleen, subjected it to gentle pressure between 2 glass plates, and viewed the preparation under a compound microscope. Parasites were identified based on morphology, infection site and previous records from fathead minnows in Alberta and Canada (e.g. McDonald and Margolis, 1995; Sandland *et al*., 2001). Infection sites are provided in Table 1.

**Table 1.** Intensity (mean ± s.d.) and prevalence (%) of parasites infecting fathead minnows (*Pimephales promelas*) from southern Alberta, Canada between 2018 and 2020.

Species	Infection site	Site						
		CC (110)	GS (121)	MQ (122)	RL (107)	SCR (105)	ST (80)	UP (110)
*Ornithodiplostomum ptychocheilus*	Brain	183.9 ± 78.9 (100%)	274.7 ± 94.9 (100%)	64.0 ± 81.0 (100%)	28.3 ± 15.4 (100%)	20.0 ± 16.8 (90%)	129.7 ± 128.2 (100%)	4.1 ± 3.3 (83%)
*Ornithodiplostomum* sp.	Body cavity	61.6 ± 53.0 (100%)	107.0 ± 84.4 (100%)	16.8 ± 34.1 (92%)	9.5 ± 6.9 (89%)	12.4 ± 12.7 (81%)	7.1 ± 4.2 (100%)	2.8 ± 2.1 (70%)
*Posthodiplostomum minimum*	Body cavity	1.2 ± 0.4 (15%)	2.1 ± 1.5 (26%)	2.4 ± 2.0 (39%)	1.5 ± 0.8 (32%)	5.8 ± 4.7 (85%)	3.9 ± 7.3 (60%)	1.2 ± 0.4 (5%)
*Diplostomum* sp.	Eye	5.9 ± 7.1 (42%)	1.3 ± 0.5 (5%)	3.5 ± 6.4 (52%)	2.7 ± 1.6 (86%)	3.6 ± 3.3 (74%)	4.9 ± 2.8 (99%)	– (0%)
*Crassiphiala bulboglossa*	Epidermis	2.1 ± 1.2 (55%)	2.0 ± 1.4 (18%)	2.4 ± 1.7 (50%)	2.3 ± 1.5 (62%)	4.8 ± 4.7 (96%)	1.4 ± 0.5 (6%)	1.1 ± 0.3 (12%)
*Contracaecum* sp.	Heart	1.4 ± 0.9 (15%)	1.5 ± 1.0 (14%)	1.6 ± 0.8 (30%)	1.1 ± 0.4 (27%)	1.2 ± 0.4 (19%)	2.4 ± 3.1 (36%)	1.2 ± 0.5 (18%)
*Goussia degiustii*	Spleen	– (62%)	– (87%)	– (95%)	– (89%)	– (90%)	– (98%)	– (67%)
*Pomphorhynchus bulbocolli*	Digestive tract	– (0%)	1.0 ± NA (1%)	1.0 ± 0.0 (3%)	– (0%)	– (0%)	1.0 ± NA (2%)	1.0 ± NA (1%)
*Ligula intestinalis*	Digestive tract	– (0%)	1.0 ± NA (1%)	1.0 ± 0.0 (2%)	1.0 ± NA (1%)	– (0%)	– (0%)	– (0%)
*Proteocephalus* sp.	Digestive tract	– (0%)	– (0%)	– (0%)	2.6 ± 2.4 (8%)	– (0%)	– (0%)	1.0 ± 0.0 (2%)

CC, Coulee Creek Stormwater Pond; GS, Gold Spring Park Pond; MQ:,McQuillan Reservoir; RL, Reesor Lake; SCR, Spruce Coulee Reservoir; ST, Stirling Lions' Fish Pond; UP, University Pond.

Intensity is not provided for eimerian *Goussia degiustii* because it was not enumerated at necropsy. Host sample size (*n*) is specified next to the sampling site.

### Terminology and treatment of data

Quantitative descriptors of parasite populations follow Bush *et al*. (1997). We considered a host population to consist of minnows sampled at a given site during a given year. Since minnow standard lengths differed among years (Kruskal–Wallis: *H* = 7.76, d.f. = 2, *P* < 0.05) and among sites (Kruskal–Wallis: *H* = 394.96, d.f. = 5, *P* < 0.001), we included standard length as a covariate in subsequent analyses. Community-level analyses were restricted to data on larval helminths from minnows collected from the 6 sites sampled in all 3 years (*n* = 675; Stirling Pond was only sampled in 2 of the 3 years). It was not possible to obtain count data for the eimerian *Goussia degiustii* or for the 5 species of myxozoan. For these species, we restrict analyses to prevalence data.

### Statistical analyses

All statistical analyses were conducted in RStudio (version 1.4.1106) running R 4.0.4 (R Core Team, 2020). We assessed annual and between-pond variation in parasite prevalence and intensity with generalized linear models (GLMs). In these models, variation in prevalence and intensity was predicted using sampling site, year and the interaction between site and year as fixed factors and minnow standard length as a covariate (prevalence/intensity – site + year + site × year + minnow standard length). Prevalence was modelled with a binomial distribution and a logit link function, whereas intensity was modelled with a negative binomial distribution and a log link function. We pruned full models using a stepwise deletion approach to remove non-significant factors (changes in model fit evaluated by likelihood-ratio test), and tested predictors in the minimum adequate models for significance. Negative binomial GLMs were run using the glm.nb function in the MASS package (Venables and Ripley, 2002), and the amount of deviance explained by the GLMs was calculated using an adjusted *D*^2^ using the Dsquared function in the modEvA package (Barbosa *et al*., 2013) in R. Due to the under-dispersion in the data for *Contracaecum* sp. counts, we used a zero-inflated negative binomial model to evaluate its abundance, rather than intensity. We ran the zero-inflated model (abundance – site + year + site × year + minnow standard length | 1) using the zeroinfl function in the pscl package (Jackman, 2020) and tested the significance of predictors with type II analysis of variance in R.

Non-metric multidimensional scaling (NMDS) was used to visualize parasite infracommunity structure. We calculated a Bray–Curtis dissimilarity matrix (based on abundance data) and a Sørensen dissimilarity matrix (based on presence–absence data) for parasite species that had ⩾10% prevalence at 1 or more sites. Uninfected fish were excluded from NMDS analysis and zero-rich abundance data were ln(x + 1)-transformed prior to analysis. The number of dimensions (k) for each ordination was chosen through an iterative process that minimized stress. We then used analysis of similarities (ANOSIM; 9999 permutations) and permutational multivariate analysis of variance (PERMANOVA; 999 permutations) to test for compositional dissimilarity among sites and years. These multivariate techniques were carried out using the metaMDS, anosim and adonis functions in the vegan package (Oksanen *et al*., 2020) in R.

We evaluated pairwise species co-occurrences in the overall sample of minnows (*n* = 675) using the probabilistic method developed by Veech (2013) and implemented in the R package cooccur (Griffith *et al*., 2016). Probabilistic methods offer an alternative to the widely employed *C*-score metric (Stone and Roberts, 1990) that is typically used as an aggregated, community-level index and may consequently obscure the co-existence of positive and negative associations within a community (Cazelles *et al*., 2016). All parasite taxa for which we had prevalence data were included in a presence–absence matrix in which parasite species were rows and individual minnows were columns. We then summarized all pairwise species associations as positive, negative or random. Positive associations were assigned to species pairs with a co-occurrence probability significantly greater than the observed frequency of co-occurrence (*P*_gt_) while negative associations were assigned to species pairs with a co-occurrence probability significantly less than the observed frequency of co-occurrence (*P*_lt_). Random associations were assigned to species pairs for which the predicted co-occurrence probability deviated from the expected co-occurrence by less than 10% of minnows.

We further tested the association between the congeners *Ornithodiplostomum* sp. and *O. ptychocheilus* at the level of the host population. To evaluate whether covariation in abundances was confounded by host length, we regressed species' abundances against minnow standard length and then tested for association by correlating the residuals. We also compared the frequency of co-occurrence at the level of individual hosts using *χ*^2^ analysis. At the within-host level, we evaluated whether minnows infected with 1 species were more or less likely to be infected with a second species using binomial generalized linear mixed models (GLMM). We recorded parasite prevalence for individual minnows as 0 (uninfected) or 1 (infected). Our GLMMs included minnow standard length as a covariate, while sampling site and year were included as random effects (prevalence_sp.1_ – prevalence_sp.2_ + standard length + [1 | site] + [1 | year]). We ran GLMMs fit by maximum likelihood using the Laplace approximation with the glmer function in the lme4 package (Bates *et al*., 2015) in R.

## Results

### General patterns of infection

**Figure 2.**
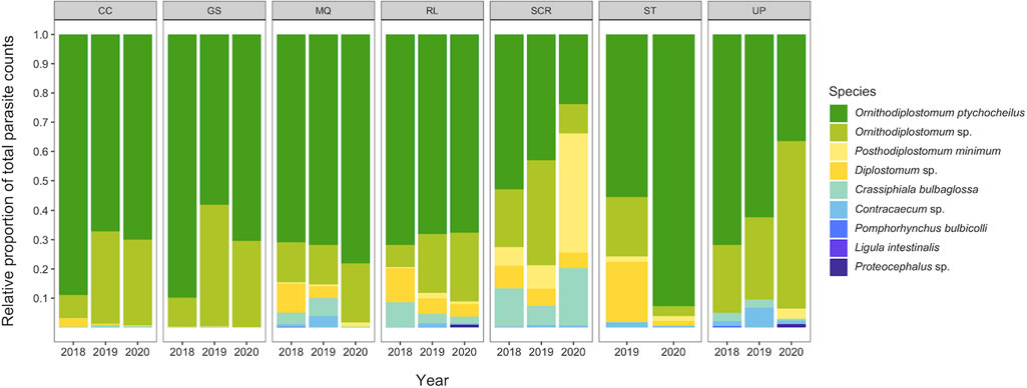
Relative abundances of parasite species recovered in fathead minnows (*Pimephales promelas*) from southern Alberta, Canada between 2018 and 2020.

We recovered 9 species of helminth, 1 species of eimerian and 5 species of myxozoan (*Myxobolus hendricksoni*, *M. hyborhynchi*, *M. rasmusseni*, *Myxobolus* sp. and *Unicauda magna*) in the total sample of 755 minnows. The prevalence and mean intensity of each of the 10 non-myxozoans, pooled across the 3 sampling years, are summarized in Table 1. The complete species by site-by-year data matrix is provided in Table S2. Ninety-nine per cent of minnows (754/755) were infected with at least 1 parasite species, and 98% (740/755) were co-infected with 2 or more species. On average, minnows were co-infected with 4.4 ± 1.4 species. Two larval trematodes, *Ornithdodiplostomum* sp. and *O. ptychocheilus*, dominated the overall parasite assemblage (Fig. 2). Together, they accounted for 101 295 of 104 945 parasites recovered (96.5%) from the total sample of 755 minnows. The maximum intensities for *Ornithdodiplostomum* sp. and *O. ptychocheilus* were 347 and 665, respectively.

Parasite prevalences and mean intensities varied significantly by site, year and the interaction between site and year (Table 2). In the GLMs, the factor ‘site’ was present and significant in all 13 minimum adequate models, while ‘year’ was significant in 12 of 13 models. The interaction term was absent from only 3 minimum adequate models (those specifying *O. ptychocheilus* prevalence, *G. degiustii* prevalence and *Posthodiplostomum minimum* intensity), but variation was attributed to site and year independently in these models. Minnow standard length positively predicted variation in prevalence and intensity. Overall, predictors in the minimum adequate models explained 19–50 and 37–89% of the variation in prevalence and intensity, respectively.

**Table 2.** Summary of associations between the prevalence and intensity of parasite species by site, year, their interaction and fathead minnow standard length using GLM analysis.

	Site		Year		Site × year		Standard length		% Deviance explained
	*χ*^2^	*P*	*χ*^2^	*P*	*χ*^2^	*P*	Estimate ± s.e.	*P*	
*Ornithodiplostomum* sp. prevalence	89.99	***	2.05	NS	81.95	***	–	–	35.3
*Ornithodiplostomum* sp. intensity	1868.95	***	285.61	***	310.18	***	0.0570 ± 0.0089	***	80.4
*O. ptychocheilus* prevalence	72.00	***	27.09	***	–	–	0.1859 ± 0.0808	*	43.2
*O. ptychocheilus* intensity	4858.70	***	52.00	***	738.50	***	0.0612 ± 0.0058	***	89.3
*P. minimum* prevalence	197.14	***	50.02	***	77.73	***	–	–	36.2
*P. minimum* intensity	151.59	***	12.35	**	–	–	0.0674 ± 0.0167	***	48.2
*Diplostomum* sp. prevalence	347.41	***	80.47	***	40.13	***	0.1197 ± 0.0423	*	50.0
*Diplostomum* sp. intensity	41.44	***	50.59	***	62.96	***	0.0862 ± 0.0154	**	37.2
*C. bulboglossa* prevalence	243.27	***	22.87	***	29.03	**	–	–	29.8
*C. bulboglossa* intensity	118.59	***	26.36	***	41.07	***	0.0293 ± 0.0127	*	39.4
*Contracaecum* sp. prevalence	13.94	*	105.36	***	26.50	**	0.0783 ± 0.0344	NS	19.3
*Contracaecum* sp. abundance	17.63	**	55.50	***	20.47	**	0.0659 ± 0.0245	*	NA^a^
*G. degiustii* prevalence	68.99	***	208.53	***	–	–	–	–	42.9

Factors absent from minimum adequate models are denoted by a dash. Only parasites with a prevalence ⩾10% were analysed. Parasite prevalence was modelled with a binomial distribution and logit link function, and intensity was modelled with a negative binomial distribution and log link function. *P* values are as follows: NS > 0.05; *<0.05; **<0.01; ***<0.001. NA: not applicable.

a *Contracaecum* sp. intensities were under-dispersed, so a zero-inflated negative binomial model was used to evaluate *Contracaecum* sp. abundance.

### Parasite infracommunity structure

**Figure 3.**
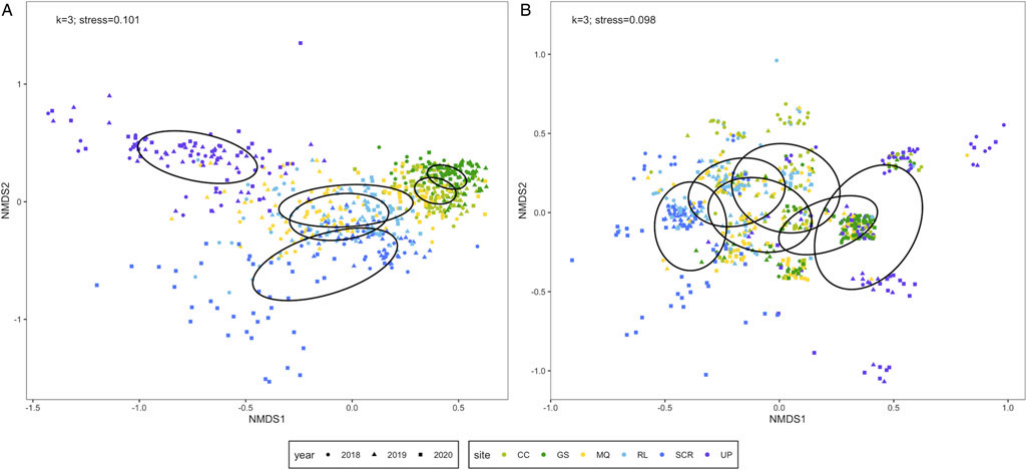
Non-metric multidimensional scaling (NMDS) ordinations of parasite infracommunities of fathead minnows (*Pimephales promelas*) from southern Alberta, Canada between 2018 and 2020. Infracommunity distances are based on (A) Bray–Curtis dissimilarities for 6 species [ln(x + 1)-transformed count data] and (B) Sørensen dissimilarities for 7 species (presence/absence data). Infracommunities are limited to taxa with ⩾10% prevalence at 1 or more sites. Ellipses represent 95% confidence intervals enclosing all points in each site. CC, Coulee Creek Stormwater Pond; GS, Gold Spring Park Pond; MQ, McQuillan Reservoir; RL, Reesor Lake; SCR, Spruce Coulee Reservoir; UP, University Pond.

The Bray–Curtis dissimilarity matrix was built from 666 infracommunities and the Sørensen dissimilarity matrix incorporated 673 infracommunities. Six species (*Ornithodiplostomum* sp., *O. ptychocheilus*, *P*. *minimum*, *Diplostomum* sp., *Crassiphiala bulboglossa* and *Contracaecum* sp.) were common to both matrices while the Sørensen matrix additionally included *G*. *degiustii*. We refer to the former matrix containing 6 species as the reduced assemblage, and the latter matrix containing 7 species as the complete assemblage. The reduced ordination had 3 dimensions and a stress value of 0.101 (Fig. 3A). Infracommunity composition differed among sites (PERMANOVA: *F*_5,660_ = 141.31, *P* < 0.001) and among years (PERMANOVA: *F*_2,663_ = 15.28, *P* < 0.001). Infracommunities were also more similar within sites/years than among sites/years (site: *R* = 0.502, *P* = 0.0001; year: *R* = 0.060, *P* = 0.0001) as indicated by ANOSIM. The complete ordination had 3 dimensions and a stress value of 0.098 (Fig. 3B). Infracommunity composition differed among sites (PERMANOVA: *F*_5,667_ = 56.14, *P* < 0.001) and among years (PERMANOVA: *F*_2,670_ = 52.10, *P* < 0.001). Infracommunities were also more similar within sites/years than among sites/years (site: *R* = 0.266, *P* = 0.0001; year: *R* = 0.122, *P* = 0.0001) as indicated by ANOSIM.

### Patterns of parasite species co-occurrence

**Figure 4.**
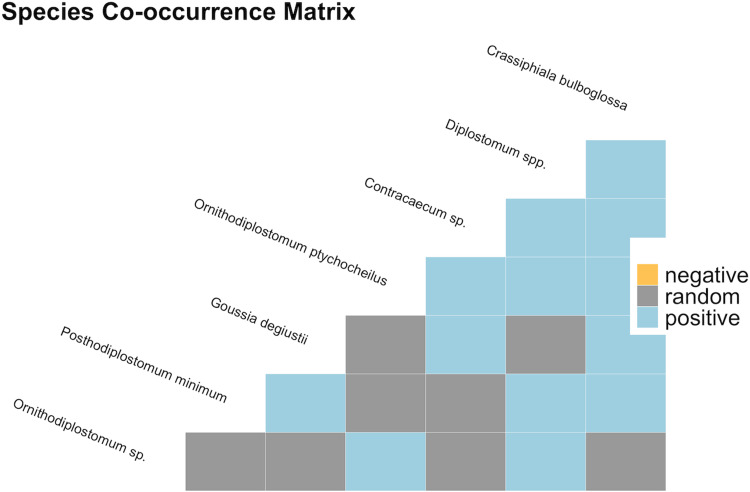
Species co-occurrence matrix for pairwise associations between parasites infecting fathead minnows (*Pimephales promelas*) from 6 sites in southern Alberta, Canada between 2018 and 2020.

The presence–absence matrix from 675 fish contained 10 parasite species, yielding 45 species pairs. Overall, most pairs (32/45; 71.1%) were classified as randomly associated. All 13 significant, non-random associations were positive (Fig. 4) indicating that they co-occurred more often than expected by chance (Table S3). The taxa with the highest and lowest number of positive associations were *C. bulboglossa* and *Diplostomum* sp. (*n* = 5) and *Ornithodiplostomum* sp. (*n* = 2), respectively. Of all possible pairs, *Ornithodiplostomum* sp. and *O. ptychocheilus* co-occurred in the greatest number of minnows (586/675; 86.8%) in the probabilistic analysis (Table S3).

**Figure 5.**
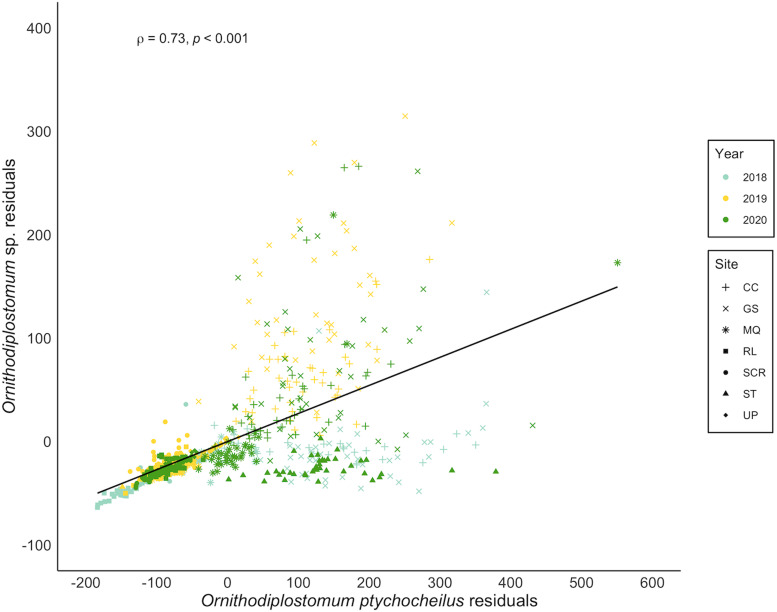
Spearman correlation between residuals from the abundance–host standard length relationships for *Ornithodiplostomum* sp. and *O*. *ptychocheilus* in fathead minnows (*Pimephales promelas*) from southern Alberta, Canada between 2018 and 2020. CC, Coulee Creek Stormwater Pond; GS, Gold Spring Park Pond; MQ, McQuillan Reservoir; RL, Reesor Lake; SCR, Spruce Coulee Reservoir; ST, Stirling Lions' Fish Pond; UP, University Pond.

**Figure 6.**
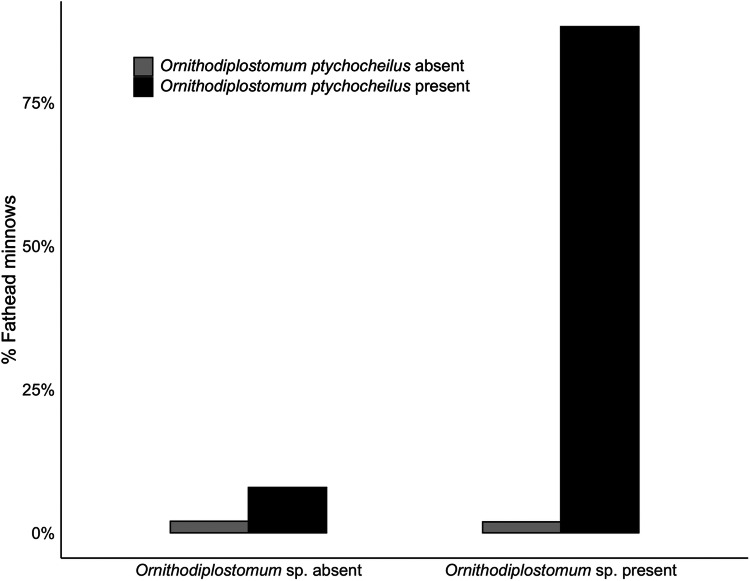
Percentage of fathead minnows (*Pimephales promelas*) infected by *Ornithodiplostomum ptychocheilus*, *Ornithodiplostomum* sp. or both species.

*Ornithodiplostomum ptychocheilus* and *Ornithodiplostomum* sp. co-occurred in 666/755 minnows (88.2%). At the population level, their mean abundances correlated positively (*n* = 20, *r* = 0.87, *P* < 0.001). Thus, when the counts of one of these species were high at a particular site in a particular year, the counts of the other species were also high at that site in that year. When covariation in interspecific abundance was examined within the 20 individual site-by-year samples, 14 were significantly positive (*r* = 0.33–0.75, 3.336 × 10^−8^ < *P* < 0.038). Within these samples of 30–40 minnows, *O. ptychocheilus* intensities were consistently higher than *Ornithodiplostomum* sp. intensities (paired *t*-test, *t* = 3.60, *P* < 0.01), with an overall mean (± s.d.) of 101.9 ± 120.1 *O. ptychocheilus* metacercariae and 32.2 ± 56.2 *Ornithodiplostomum* sp. metacercariae. When covariation in intensities was examined at the level of individual hosts, independently of host length, this relationship persisted (Fig. 5; *n* = 755, *r* = 0.73, *P* < 0.001). Additionally, *Ornithodiplostomum* sp. and *O. ptychocheilus* were significantly more likely to co-occur in minnows than expected by chance (Fig. 6; Pearson's *χ*^2^ = 1616.3, *P* < 0.001) so that the presence of 1 species positively predicted co-occurrence of the other (*Ornithodiplostomum* sp.–*O. ptychocheilus*: *z* = 3.34, *P* < 0.001; *O. ptychocheilus*–*Ornithodiplostomum* sp.: *z* = 2.41, *P* < 0.05).

## Discussion

Patterns of infection for larvae of the digenean trematodes *Ornithodiplostomum* sp. and *O. ptychocheilus* are distinct from the rest of the parasite assemblage in minnows. The 2 species were numerically dominant, together accounting for over 95% of the >100 000 parasites counted. Their mean and maximum intensities exceeded those of the next most dominant species by an order of magnitude at some sites. Additionally, they were the only 2 helminth species present at all sites in all years and they co-occurred in more minnows than any other species pair. Finally, both the occurrences and intensities of these 2 species were significantly positively associated within individual fish, within populations of fish and within the total sample of 755 fish. Although the non-equilibrial and unstructured nature of parasite communities in fish is frequently emphasized in the literature (Kennedy, 2009; Marcogliese and Goater, 2016), the *Ornithodiplostomum* species couplet is a repeatable and predictable component of the overall parasite community in fathead minnows. Our results indicate that 2-year-old adult fathead minnows collected from lentic habitats will almost always contain encysted *O. ptychocheilus* and *Ornithodiplostomum* sp. in their optic lobes and livers, respectively. This pattern appears to be consistent between years and between geographically separated waterbodies located within the northern distribution of this host.

Sets of congeners have been reported to dominate parasite assemblages in other host species (Kennedy and Bush, 1992; Poulin, 1999). Well-known examples include dactylogyrid trematodes on fish, strongyloid nematodes in horses and digenean eye flukes in freshwater fish. Indeed, our identification of the *Ornithodiplostomum* species couplet likely underestimates the overall dominance of this group of larval strigeid trematodes in fathead minnows. Molecular sequencing of these and related trematodes led Achatz *et al*. (2021) to synonymize the genera *Ornithodiplostomum* and *Posthodiplostomum*. Should this revised taxonomic scheme hold, then *O. ptychocheilus* (now *P. ptychocheilus*; Achatz *et al*., 2021), *Ornithodiplostomum* sp. (now *Posthodiplostomum* sp.) and *P. minimum* would comprise a congeneric triplet that dominates minnows collected from lentic waterbodies on the Great Plains. Including *P. minimum* within this proposed set of dominant species also expands the geographical scope in which larval *Ornithodiplostomum/Posthodiplostomum* dominates to include samples of minnows from southern Alberta (this study), northern Alberta (Sandland *et al*., 2001) and central Minnesota (Wisenden *et al*., 2012).

Both positive and random pairwise associations occur among the parasite species that co-infect fathead minnows. These associations occurred within individual hosts and within samples of hosts. Positive patterns of co-occurrence illustrate that infections are often not independent of one another. Positive associations can arise if certain hosts represent higher-quality patches of habitat (Krasnov *et al*., 2011), if one species facilitates infection for a second species, or if species share common intermediate hosts or modes of transmission. Although the trematode species found in fatheads do not all share a common first intermediate host, their lymnaeid, physid and planorbid snail hosts tend to co-occur within the nutrient-rich littoral zones of southern Alberta lakes and ponds. The positive associations between species such as *Ornithodiplostomum* sp. and *Diplostomum* sp. and *Crassiphiala bulboglossa* and *Diplostomum* sp., each of which utilizes different snail intermediate hosts, can probably best be explained by their use of common, co-occurring and annual snails in these sites. Notably, we did not detect negative associations within the overall parasite community. Negative associations arise primarily from competition, for example, between species that infect the same host tissue, although they are typically outnumbered by positive associations in many host–parasite systems (Lotz and Font, 1991; Krasnov *et al*., 2011). The absence of negative pairwise associations suggests that interspecific competition is either not present among the parasites of fatheads or that this process is undetectable with our approach.

Within well-characterized parasite communities, positive covariation in helminth counts is more common than negative or neutral covariation, especially for congeners (e.g. Bucknell *et al*., 1996). Positive covariation in worm counts can arise when co-infecting parasites share routes and rates of transmission. For metacercariae within second intermediate hosts, covariation in occurrence and intensity can thus be expected for co-infecting species that share the same snail intermediate host (Karvonen *et al*., 2006; Faltýnková *et al*., 2011; Lagrue and Poulin, 2015). The strength and direction of the association between *Ornithodiplostomum* sp. and *O. ptychocheilus* likely stems from their shared use of the physid pond snails, *Physa gyrina* and *P. integra* (Matisz *et al*., 2010; Matisz and Goater, 2010). *Posthodiplostomum minimum* originating from fathead minnows also used *P. gyrina* (Schleppe and Goater, 2004). *Physa gyrina* and *P. integra* are sympatric and syntopic in many waterbodies in south-central Canada (Pip and Franck, 2008). Barcoding results involving cercariae sequenced from 5 species of pond snail collected from ponds/lakes in central Alberta identified 2 species of *Ornithodiplostomum* in *P. gyrina*, neither of which was found in any other species of snail (Gordy *et al*., 2016). This combination of empirical and molecular evidence indicates that the congeneric parasites that dominate fathead minnow communities in ponds in southern Alberta and whose occurrences and intensities are strongly positively correlated likely share physid intermediate hosts.

The results of our GLMs showed that site, year and their interactions were significant predictors of interspecific prevalences and intensities. The significance of the interaction between site and year in all minimum adequate models in which it appeared highlights that between-pond variation was dependent on annual variation. For instance, it was common to observe 5–10-fold differences in metacercariae intensities between sites and between years. Whereas mean *O. ptychocheilus* intensities in minnows in Goldspring Pond were generally consistent between the 3 years (ca. 250–300 metacercariae/host), they varied erratically between <50 and almost 200 for *Ornithodiplostomum* sp. over the same period. There was especially striking variation in mean intensities in samples of minnows from McQuillan Pond over this period. Variation in *O. ptychocheilus* intensities in minnows collected from 4 natural lakes in north-central Alberta were attributed to lake, sampling year and their interactions (Sandland *et al*., 2001). These results emphasize that local, site-level characteristics that vary between years [e.g. lake surface area (Rossiter and Davidson, 2018), lake bottom type (Ondračková *et al*., 2004) and water temperature (Karvonen *et al*., 2013)] are likely important determinants of cercariae transmission rates between snail and fish intermediate hosts (Pietrock and Marcogliese, 2003). These factors appear to play important roles in transmission in both minnows in natural lakes (Sandland *et al*., 2001; Goater and Wisenden, unpublished observations) and in artificial reservoirs.

A key objective of the current study was to identify pairs of species that regularly co-infect individual minnows. Should such pairs exist, they would make logical candidates for follow-up reciprocal exposure trials to test hypotheses regarding the consequences of co-infection (e.g. Karvonen *et al*., 2009; Johnson and Buller, 2011). Our results point to the potential of the *Ornithodiplostomum* species couplet as an appropriate model. Not only did the 2 species consistently co-occur within a large sample of minnows, but co-occurrence was highly predictable between sites and years. Given that both species are amenable to experimental manipulation in a laboratory setting (Matisz *et al*., 2010; Matisz and Goater, 2010), the results of this study indicate that the fathead minnow–*Ornithodiplostomum* spp. interaction provides an ideal model for experimental tests of the consequences of coinfection on both parasite and host performance.

## Supporting information

Hirtle et al. supplementary material 1 Hirtle et al. supplementary material

Hirtle et al. supplementary material 2 Hirtle et al. supplementary material

Hirtle et al. supplementary material 3 Hirtle et al. supplementary material

## Data Availability

The data are available from the corresponding author upon reasonable request.

## Supplementary material

The supplementary material for this article can be found at https://doi.org/10.1017/S0031182023000859
