# Supplementary material for: A congeneric and non-randomly associated pair of larval trematodes dominates the assemblage of co-infecting parasites in fathead minnows (Pimephales promelas)
Source: Parasitology. 2023 Sep 14;150(11):1006–14. doi: 10.1017/S0031182023000859 (PMC10941217; doi:10.1017/S0031182023000859)
Supplement: Hirtle et al. supplementary material 1 — Hirtle et al. supplementary material [file S0031182023000859sup001.docx]

Table S1. Geographic coordinates of sampling sites expressed in decimal degrees.

| Sampling site | Latitude | Longitude |
| --- | --- | --- |
| Coulee Creek Stormwater Pond | 49.656625 | -112.784628 |
| Gold Spring Park Pond | 49.096131 | -111.995817 |
| McQuillan Reservoir | 49.647114 | -112.459358 |
| Reesor Lake | 49.664333 | -110.105672 |
| Spruce Coulee Reservoir | 49.672761 | -110.180889 |
| Stirling Lions’ Fish Pond | 49.500775 | -112.536664 |
| University Pond | 49.680528 | -112.870672 |
