# Supplementary material for: A congeneric and non-randomly associated pair of larval trematodes dominates the assemblage of co-infecting parasites in fathead minnows (Pimephales promelas)
Source: Parasitology. 2023 Sep 14;150(11):1006–14. doi: 10.1017/S0031182023000859 (PMC10941217; doi:10.1017/S0031182023000859)
Supplement: Hirtle et al. supplementary material 2 — Hirtle et al. supplementary material [file S0031182023000859sup002.docx]

Table S2. Prevalence (%) and mean abundance (SD; range) of parasites infecting fathead minnows (*Pimephales promelas*) from southern Alberta, Canada between 2018–2020. NE: not enumerated; NS: not sampled. CC: Coulee Creek Stormwater Pond; GS: Gold Spring Park Pond; MQ: McQuillan Reservoir; RL: Reesor Lake; SCR: Spruce Coulee Reservoir; ST: Stirling Lions’ Fish Pond; UP: University Pond.

| Site | Parasite species | Prevalence (*n*) | | | Mean abundance (SD; range) | | |
| --- | --- | --- | --- | --- | --- | --- | --- |
|  |  | 2018 | 2019 | 2020 | 2018 | 2019 | 2020 |
| CC | *Ornithodiplostomum ptychocheilus* | 100.0 (30) | 100.0 (40) | 100.0 (40) | 208.63 (101.9; 70-433) | 187.5 (61.5; 69-370) | 161.75 (66.5; 59-319) |
|  | *Ornithodiplostomum* sp. | 100.0 (30) | 100.0 (40) | 100.0 (40) | 18.47 (10.4; 2-46) | 87.88 (39.2; 29-203) | 67.65 (62.6; 6-295) |
|  | *Posthodiplostomum minimum* | 13.3 (30) | 0.0 (40) | 32.5 (40) | 0.17 (0.5; 0-2) | – | 0.4 (0.6; 0-2) |
|  | *Diplostomum* sp. | 73.3 (30) | 47.5 (40) | 12.5 (40) | 6.63 (8.5; 0-25) | 1.5 (2.4; 0-10) | 0.28 (0.9; 0-6) |
|  | *Crassiphiala bulboglossa* | 43.3 (30) | 72.5 (40) | 47.5 (40) | 0.67 (0.8; 0-2) | 1.65 (1.4; 0-5) | 1 (1.4; 0-5) |
|  | *Contracaecum* sp. | 0.0 (30) | 40.0 (40) | 2.5 (40) | – | 0.55 (0.9; 0-4) | 0.03 (0.2; 0-1) |
|  | *Goussia degiustii* | 3.3 (30) | 82.5 (40) | 85.0 (40) | NE | NE | NE |
|  | *Pomphorhynchus bulbocolli* | 0.0 (30) | 0.0 (40) | 0.0 (40) | – | – | – |
|  | *Ligula intestinalis* | 0.0 (30) | 0.0 (40) | 0.0 (40) | – | – | – |
|  | *Proteocephalus* sp. | 0.0 (30) | 0.0 (40) | 0.0 (40) | – | – | – |
| GS | *O. ptychocheilus* | 100.0 (41) | 100.0 (40) | 100.0 (40) | 313.02 (101.7; 115-529) | 254.13 (76.2; 74-465) | 255.9 (91.1; 134-585) |
| GS | *Ornithodiplostomum* sp. | 100.0 (41) | 100.0 (40) | 100.0 (40) | 34.68 (34.9; 1-185) | 181.43 (72.3; 61-347) | 106.55 (63.0; 13-295) |
|  | *P. minimum* | 17.1 (41) | 42.5 (40) | 20.0 (40) | 0.27 (0.7; 0-3) | 0.95 (1.3; 0-5) | 0.48 (1.4; 0-8) |
|  | *Diplostomum* sp. | 0.0 (41) | 15.0 (40) | 0.0 (40) | – | 0.2 (0.5; 0-2) | – |
|  | *C. bulboglossa* | 22.0 (41) | 27.5 (40) | 5.0 (40) | 0.54 (1.3; 0-7) | 0.5 (0.9; 0-4) | 0.05 (0.2; 0-1) |
|  | *Contracaecum* sp. | 0.0 (41) | 32.5 (40) | 10.0 (40) | – | 0.43 (0.7; 0-2) | 0.2 (0.8; 0-5) |
|  | *G. degiustii* | 65.9 (41) | 95.0 (40) | 100.0 (40) | NE | NE | NE |
|  | *P. bulbocolli* | 0.0 (41) | 0.0 (40) | 2.5 (40) | – | – | 0.03 (0.2; 0-1) |
|  | *L. intestinalis* | 2.4 (41) | 0.0 (40) | 0.0 (40) | 0.02 (0.2; 0-1) | – | – |
|  | *Proteocephalus* sp. | 0.0 (41) | 0.0 (40) | 0.0 (40) | – | – | – |
| MQ | *O. ptychocheilus* | 100.0 (42) | 100.0 (40) | 100.0 (40) | 29.98 (35.0; 5-161) | 18.5 (7.8; 5-39) | 145.23 (92.8; 69-665) |
|  | *Ornithodiplostomum* sp. | 92.9 (42) | 82.5 (40) | 100.0 (40) | 5.81 (8.4; 0-39) | 3.48 (3.1; 0-11) | 37.6 (49.8; 7-263) |
|  | *P. minimum* | 16.7 (42) | 15.0 (40) | 87.5 (40) | 0.17 (0.4; 0-1) | 0.18 (0.4; 0-2) | 2.55 (2.1; 0-9) |
|  | *Diplostomum* sp. | 71.4 (42) | 67.5 (40) | 17.5 (40) | 4.21 (7.7; 0-33) | 1.03 (0.4; 0-4) | 0.23 (0.5; 0-2) |
|  | *C. bulboglossa* | 69.0 (42) | 55.0 (40) | 25.0 (40) | 1.69 (1.7; 0-6) | 1.6 (2.1; 0-9) | 0.33 (0.6; 0-3) |
|  | *Contracaecum* sp. | 19.0 (42) | 60.0 (40) | 10.0 (40) | 0.33 (0.8; 0-3) | 1 (1; 0-3) | 0.1 (0.3; 0-1) |
|  | *G. degiustii* | 88.1 (42) | 97.5 (40) | 100.0 (40) | NE | NE | NE |
| MQ | *P. bulbocolli* | 7.1 (42) | 0.0 (40) | 2.5 (40) | 0.07 (0.3; 0-1) | – | 0.03 (0.2; 0-1) |
|  | *L. intestinalis* | 4.8 (42) | 0.0 (40) | 0.0 (40) | 0.05 (0.2; 0-1) | – | – |
|  | *Proteocephalus* sp. | 0.0 (42) | 0.0 (40) | 0.0 (40) | – | – | – |
| RL | *O. ptychocheilus* | 100.0 (30) | 100.0 (37) | 100.0 (40) | 13 (6.6; 2-26) | 29.38 (12.4; 11-60) | 38.9 (12.7; 13-63) |
|  | *Ornithodiplostomum* sp. | 63.3 (30) | 97.3 (37) | 100.0 (40) | 1.37 (1.6; 0-7) | 8.68 (4.9; 0-23) | 13.5 (6.9; 3-32) |
|  | *P. minimum* | 6.7 (30) | 51.4 (37) | 32.5 (40) | 0.07 (0.2; 0-1) | 0.78 (0.9; 0-4) | 0.53 (0.9; 0-3) |
|  | *Diplostomum* sp. | 86.7 (30) | 83.8 (37) | 87.5 (40) | 2.1 (1.5; 0-6) | 2.29 (1.4; 0-6) | 2.48 (2.1; 0-8) |
|  | *C. bulboglossa* | 73.3 (30) | 51.4 (37) | 62.5 (40) | 1.53 (1.4; 0-5) | 1.38 (1.9; 0-9) | 1.3 (1.3; 0-4) |
|  | *Contracaecum* sp. | 3.3 (30) | 51.4 (37) | 22.5 (40) | 0.03 (0.2; 0-1) | 0.59 (0.6; 0-2) | 0.25 (0.5; 0-2) |
|  | *G. degiustii* | 60.0 (30) | 100.0 (37) | 100.0 (40) | NE | NE | NE |
|  | *P. bulbocolli* | 0.0 (30) | 0.0 (37) | 0.0 (40) | – | – | – |
|  | *L. intestinalis* | 0.0 (30) | 2.7 (37) | 0.0 (40) | – | 0.03 (1.2; 0-1) | – |
|  | *Proteocephalus* sp. | 0.0 (30) | 0.0 (37) | 22.5 (40) | – | – | 0.58 (1.5; 0-8) |
| SCR | *O. ptychocheilus* | 100.0 (30) | 100.0 (35) | 75.0 (40) | 32.87 (16.7; 5-94) | 21.77 (12.1; 5-65) | 3.73 (6.3; 0-30) |
|  | *Ornithodiplostomum* sp. | 100.0 (30) | 100.0 (35) | 50.0 (40) | 12.1 (14.5; 1-84) | 18.03 (11.1; 6-60) | 1.58 (2.3; 0-7) |
|  | *P. minimum* | 66.7 (30) | 91.4 (35) | 92.5 (40) | 3.97 (5.2; 0-23) | 4.06 (3.3; 0-14) | 6.35 (5.2; 0-25) |
| SCR | *Diplostomum* sp. | 96.7 (30) | 94.3 (35) | 40.0 (40) | 4.8 (4.3; 0-21) | 2.94 (2.4; 0-9) | 0.83 (1.2; 0-5) |
|  | *C. bulboglossa* | 100.0 (30) | 100.0 (35) | 90.0 (40) | 8.1 (5.9; 1-36) | 3.37 (2.2; 1-9) | 3.08 (3.7; 0-20) |
|  | *Contracaecum* sp. | 16.7 (30) | 34.3 (35) | 7.5 (40) | 0.2 (0.5; 0-2) | 0.4 (0.6; 0-2) | 0.1 (0.4; 0-2) |
|  | *G. degiustii* | 66.7 (30) | 100.0 (35) | 100.0 (40) | NE | NE | NE |
|  | *P. bulbocolli* | 0.0 (30) | 0.0 (35) | 0.0 (40) | – | – | – |
|  | *L. intestinalis* | 0.0 (30) | 0.0 (35) | 0.0 (40) | – | – | – |
|  | *Proteocephalus* sp. | 0.0 (30) | 0.0 (35) | 0.0 (40) | – | – | – |
| ST | *O. ptychocheilus* | NS | 100.0 (40) | 100.0 (40) | NS | 13.88 (7.1; 3-31) | 245.6 (74.6; 134-516) |
|  | *Ornithodiplostomum* sp. |  | 100.0 (40) | 100.0 (40) |  | 5.03 (2.6; 1-12) | 9.08 (4.4; 2-23) |
|  | *P. minimum* |  | 37.5 (40) | 82.5 (40) |  | 0.48 (0.7; 0-2) | 4.23 (7.9; 0-49) |
|  | *Diplostomum* sp. |  | 100.0 (40) | 97.5 (40) |  | 5.13 (2.8; 1-13) | 4.55 (2.9; 0-10) |
|  | *C. bulboglossa* |  | 5.0 (40) | 7.5 (40) |  | 0.05 (0.2; 0-1) | 0.13 (0.5; 0-2) |
|  | *Contracaecum* sp. |  | 32.5 (40) | 40.0 (40) |  | 0.43 (0.7; 0-3) | 1.33 (2.9; 0-15) |
|  | *G. degiustii* |  | 97.5 (40) | 97.5 (40) |  | NE | NE |
|  | *P. bulbocolli* |  | 0.0 (40) | 2.5 (40) |  | – | 0.03 (0.2; 0-1) |
|  | *L. intestinalis* |  | 0.0 (40) | 0.0 (40) |  | – | – |
| ST | *Proteocephalus* sp. | NS | 0.0 (40) | 0.0 (40) | NS | – | – |
| UP | *O*. *ptychocheilus* | 86.7 (30) | 95.0 (40) | 67.5 (40) | 4.33 (4.1; 0-14) | 4.43 (3.0; 0-13) | 1.55 (1.8; 0-7) |
|  | *Ornithodiplostomum* sp. | 70.0 (30) | 67.5 (40) | 72.5 (40) | 1.4 (1.2; 0-4) | 2 (2.3; 0-10) | 2.43 (2.5; 0-9) |
|  | *P*. *minimum* | 0.0 (30) | 0.0 (40) | 12.5 (40) | – | – | 0.15 (0.4; 0-2) |
|  | *Diplostomum* sp. | 0.0 (30) | 0.0 (40) | 0.0 (40) | – | – | – |
|  | *C*. *bulboglossa* | 13.3 (30) | 20.0 (40) | 2.5 (40) | 0.17 (0.5; 0-2) | 0.2 (0.4; 0-1) | 0.03 (0.2; 0-1) |
|  | *Contracaecum* sp. | 10.0 (30) | 37.5 (40) | 5.0 (40) | 0.1 (0.3; 0-1) | 0.48 (0.7; 0-3) | 0.05 (0.2; 0-1) |
|  | *G*. *degiustii* | 13.3 (30) | 85.0 (40) | 90.0 (40) | NE | NE | NE |
|  | *P*. *bulbocolli* | 3.3 (30) | 0.0 (40) | 0.0 (40) | 0.03 (0.2; 0-1) | – | – |
|  | *L*. *intestinalis* | 0.0 (30) | 0.0 (40) | 0.0 (40) | – | – | – |
|  | *Proteocephalus* sp. | 0.0 (30) | 0.0 (40) | 5.0 (40) | – | – | 0.05 (0.2; 0-1) |
