# Supplementary material for: A congeneric and non-randomly associated pair of larval trematodes dominates the assemblage of co-infecting parasites in fathead minnows (Pimephales promelas)
Source: Parasitology. 2023 Sep 14;150(11):1006–14. doi: 10.1017/S0031182023000859 (PMC10941217; doi:10.1017/S0031182023000859)
Supplement: Hirtle et al. supplementary material 3 — Hirtle et al. supplementary material [file S0031182023000859sup003.docx]

Table S3. Number of observed and expected co-occurrences for significant, non-random species associations between parasites infecting fathead minnows (*Pimephales promelas*) from southern Alberta, Canada.

Minnows from sites sampled in all three years (*n* = 675 minnows) were included in the analysis. *P*_lt_ and *P*_gt_ represent the probability that each species pair co-occurs less than or greater than expected by chance, respectively. Cb: *Crassiphiala bulboglossa;* Ct: *Contracaecum* sp.; Ds: *Diplostomum* sp.; Gd: *Goussia degiustii;* Op: *Ornithodiplostomum ptychocheilus*; Osp: *Ornithodiplostomum* sp.; Pm: *Posthodiplostomum minimum*.

| Species 1 | Species 2 | Minnows with species 1 | Minnows with species 2 | Observed # of minnows with both species | Probability both species co-occur | Expected # of minnows with both species | *P*_lt_ | *P*_gt_ |
| --- | --- | --- | --- | --- | --- | --- | --- | --- |
| Op | Osp | 646 | 600 | 586 | 0.851 | 574.2 | 1.00000 | 0.00000 |
| Op | Ds | 646 | 286 | 284 | 0.406 | 273.7 | 1.00000 | 0.00002 |
| Op | Cb | 646 | 324 | 316 | 0.459 | 310.1 | 0.99355 | 0.01850 |
| Op | Ct | 646 | 139 | 138 | 0.197 | 133.0 | 0.99894 | 0.00949 |
| Osp | Ds | 600 | 286 | 263 | 0.377 | 254.2 | 0.99012 | 0.01903 |
| Pm | Ds | 225 | 286 | 117 | 0.141 | 95.3 | 0.99987 | 0.00024 |
| Pm | Cb | 225 | 324 | 134 | 0.160 | 108.0 | 0.99999 | 0.00001 |
| Pm | Gd | 225 | 553 | 208 | 0.273 | 184.3 | 1.00000 | 0.00000 |
| Ds | Cb | 286 | 324 | 197 | 0.203 | 137.3 | 1.00000 | 0.00000 |
| Ds | Ct | 286 | 139 | 78 | 0.087 | 58.9 | 0.99991 | 0.00018 |
| Cb | Ct | 324 | 139 | 79 | 0.099 | 66.7 | 0.99257 | 0.01239 |
| Cb | Gd | 324 | 553 | 278 | 0.393 | 265.4 | 0.99569 | 0.00767 |
| Ct | Gd | 139 | 553 | 128 | 0.169 | 113.9 | 0.99995 | 0.00016 |
